# A Case of Gastric Intramural Hematoma Caused by Anisakis Infection

**DOI:** 10.1155/2020/9260318

**Published:** 2020-06-26

**Authors:** Sang Jin Lee

**Affiliations:** Department of Internal Medicine, University of Ulsan College of Medicine, Gangneung Asan Hospital, Gangneung, Republic of Korea

## Abstract

A sixty-year-old lady admitted complaining of epigastric pain and hematemesis. On admission, esophagogastroduodenoscopic examination revealed ruptured intramural hematoma on the antrum of stomach. Eight days later, follow-up EGD showed improving ruptured intramural hematoma and one anisakis larva. Therefore, the gastric intramural hematoma was considered to be caused by anisakis infection. She recovered after ten days of conservative treatment.

## 1. Introduction

Intramural hematoma of the gastrointestinal tract is a rare disease. In the upper gastrointestinal tract, most of intramural hematomas are localized either in the esophagus or in the duodenum. Gastric intramural hematoma was rarely found [1]. It can result from recurrent vomiting, endoscopic therapy, peptic ulcer disease, and trauma. It is associated with coagulopathy and anticoagulation therapy [2, 3].

Anisakis infection can occur due to ingestion of raw fish or undercooked seafood. The raw fish includes pollack, sea eel, croaker, conger, long-legged octopus, and squid. The most common symptom of anisakiasis is abdominal pain. Nausea and vomiting, hematemesis and melena, and chest discomfort are followed by abdominal pain. Endoscopic findings of anisakis infection are worms, mucosal edema, erythema, pseudotumor, mucosal fold thickening, and mucosal erosion [4].

A case of gastric intramural hematoma caused by anisakis infection is very rare. Therefore, we report this case with a review of literature.

## 2. Case Report

A 60-year-old female visited our emergency department complaining of epigastric pain and hematemesis. She ate sliced raw fish one day ago. She had hypertension. There was no noteworthy fact in her social and family history. She did not take any antiplatelet drug and anticoagulation drug. On her initial visit, blood pressure was 105/91 mmHg, pulse rate was 72 beats per minute, respiratory rate was 24 breaths per minute, and body temperature was 37.2°C. In abdominal physical examination, there was pain and tenderness in the epigastric area. But there was no rebound tenderness and muscle rigidity. The bowel sound was normal. In laboratory test, the white blood cell count was 14,900/mm^3^, hemoglobin was 10 g/dL, and platelet count was 199,000/mm^3^. The result of coagulation test was PT 1.13 (INR) and aPTT 24.4 sec. The result of blood chemistry was total protein 5.9 g/dL, albumin 3.5 g/dL, AST 27 IU/L, ALT 11 IU/L, ALP 118 IU/L, total bilirubin 0.6 mg/dL, BUN 21.2 mg/dL, creatinine 0.8 mg/dL, Na 142 mEq/L, K 3.8 mEq/L, Cl 106 mmol/L, CRP 0.08 mg/dL, and blood sugar 145 mg/dL.

**Figure 1:**
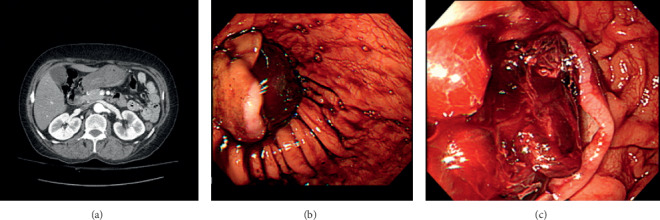
(a) Abdominal computed tomography scan showing about 8 × 3 cm nonenhancing mass (50∼60 Hounsfield unit) in the stomach antrum and lower body, suggesting submucosal hematoma. (b) Gastroscopy showing ruptured intramural hematoma on the anterior wall of antrum and low body. (c) Gastroscopy showing ruptured mucosa and hematoma in inside the mucosa wall.

Abdominal computed tomography scan showed about 8 × 3 cm nonenhancing mass in the stomach antrum and lower body. The Hounsfield unit of mass was 50∼60 units (Figure 1(a)). It suggested submucosal hematoma in stomach.

Emergent esophagogastroduodenoscopy was performed. A pool of blood was on the greater curvature side of body in the stomach. Greater than 5 cm-sized mass was noted on the anterior wall of body and antrum. The mass was round, dark brownish. But surrounding mucosa was normal. The overlying mucosa of mass was ruptured. Dark red hematoma was noted inside the mass (Figure 1(b)). Removal of hematoma and hemostasis could not be performed owing to noncooperation of the patient.

She was diagnosed with ruptured intramural hematoma. We decided to treat conservatively because further bleeding was not occurred. We treated with intravenous proton pump inhibitor and fluids. In addition, we made her fasting for one week.

**Figure 2:**
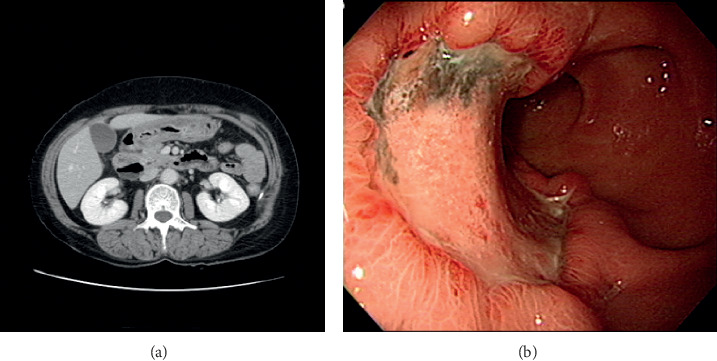
(a) Significant decreased size of hematoma in the stomach antrum and lower body compared with previous computed tomography. No visible active bleeding in stomach is observed. (b) Gastroscopy showing active ulcer on the anterior wall of the antrum and low body.

**Figure 3:**
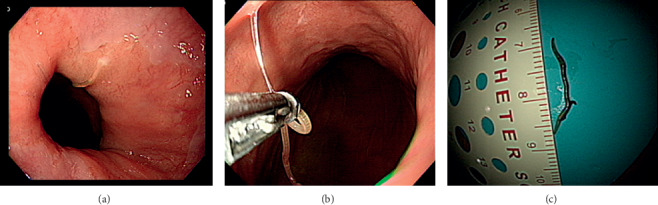
(a) Gastroscopy showing whitish thread-like worm on gastroesophageal junction. (b) Gastroscopic finding: the whitish thread-like worm is being removed by using a biopsy forcep. (c) Gross finding showing 2 cm whitish thread-like worm, identified as anisakis larva.

On hospital day 8, the gastric intramural hematoma has disappeared in the follow-up abdominal CT scan (Figure 2(a)). On follow-up EGD, the gastric intramural hematoma on anterior wall of low body and proximal antrum has disappeared. EGD showed active ulcer on the same site (Figure 2(b)). At that time, EGD showed a whitish worm resembling a short thread. The worm moved at the gastroesophageal junction. The worm was removed out of the body by using a biopsy forcep. The worm was a 2 cm-sized nematode. It was confirmed as anisakis (Figures 3(a)–3(c)).

Then, the meal was served. Further bleeding was not occurred. The patient was recovered and discharged without other complications.

## 3. Discussion

Intramural hematoma of the gastrointestinal tract is a rare disease. It can result from recurrent vomiting, endoscopic therapy, peptic ulcer disease, and trauma. It is associated with coagulopathy and anticoagulation therapy. The case of spontaneous duodenal intramural hematoma was reported in Henoch–Schönlein purpura. The case of biopsy-induced gastric intramural hematoma was reported. The case of epinephrine injection-induced intramural hematoma in liver cirrhosis was reported [5]. Rohrer et al. reported four cases of duodenal hematoma and one case of gastric hematoma in 227 bleeding peptic ulcer [3].

Anisakiasis is becoming clinical and epidemiological problem owing to the changes in diet style. Anisakis is a genus of parasitic nematodes that have lifecycles involving fish and marine mammals. Anisakiasis can be occurred due to ingestion of raw fish or undercooked seafood. The rates of anisakis infection are 68% in the stomach and 30% in the small intestine. Symptoms of anisakiasis are sudden epigastric pain, nausea, and vomiting. From two to ten hours after eating raw fish and seafood, the symptoms develop.

Intramural esophagogastric hematoma in hemophilia caused by tuberculosis infection was reported [6]. However, a report of gastric intramural hematoma caused by anisakis infection has not been published. To our knowledge, this case is the first English-written report of gastric intramural hematoma caused by anisakis infection. Anisakis larva goes through mucosa and muscle and can injure submucosal vessels. Bleeding from injured submucosal vessels can lead to intramural hematoma.

In this case, the location of anisakis and intramural hematoma was different. Intramural hematoma was at the anterior wall of body and antrum. But anisakis larva was at the gastroesophageal junction. Anisakis larva can move around inside the stomach. Therefore, the location of anisakis and intramural hematoma may be different.

Gastric intramural hematoma secondary to coagulopathy and anticoagulation therapy are treated conservatively. Conservative treatment is based on resolution of intramural hematoma due to high vascularity in the submucosa. In Vivek Dhawan et al. 's report, six reported cases of gastric hematoma secondary to hemophilia were managed with transfusion. One of them died from ongoing hemorrhage. Four cases related to anticoagulation therapy were treated conservatively. One of the four cases underwent arterial embolization [7]. If further bleeding is noted during conservative management, surgical approach is considered. In this case, the patients were recovered through conservative treatment in a week.

In summary, we reported a case of gastric intramural hematoma caused by anisakis infection. Gastric intramural hematoma may be one of the symptoms associated with anisakis infection.
